# Re: The effect of urethroplasty surgery on erectile and orgasmic functions: a prospective study

**DOI:** 10.1590/S1677-5538.IBJU.2018.0793

**Published:** 2019-04-01

**Authors:** Michael S. Floyd, Ahmad M. Omar, Andrew D. Baird, Paul C. B. Anderson

**Affiliations:** 1 Department of Reconstructive Urology, St Helens & Knowsley Hospital NHS Trust, Whiston Hospital, Warrington Road, Liverpool, United Kingdom;; 2 Department of Reconstructive Urology, Aintree University Hospital Lower Lane, Aintree, Liverpool, United Kingdom;; 3 Department of Genitourethral Reconstruction, Russell's Hall Hospital Pensnett Road, Dudley, West Midlands, United Kingdom

*To the editor,*

We read with interest the recent paper by Urkmez et al examining the effects of urethroplasty on erectile and orgasmic function (1).

The authors report a prospective study involving 60 males who underwent buccal graft substitution, penile skin flap or anastomotic urethroplasty by a single surgeon and report the outcomes on both erectile and orgasmic function using the international index of erectile function (IIEF) score.

The authors conclude by stating that urethroplasty does not affect erectile, sexual or orgasmic function irrespective of surgery type, stricture location or length but acknowledge the limitations of the study including the lack of validated questionnaires.

This study does highlight one of the challenges for urethral surgeons: measuring sexual outcomes following urethroplasty. As endoscopic management of recurrent stricture disease moves towards formal urethroplasty with better long term success rates, assessing outcomes remains paramount from a patient and surgical perspective. To date, there have been limited attempts at developing specific patient reported outcomes (PROMS) for anterior urethral reconstruction (2). The first study performed to evaluate urethroplasty outcomes with a validated PROM did not assess sexual or erectile function (3). There remains a paucity of validated questionnaires available to the urethral surgeon to assess sexual function post urethroplasty. The IIEF and its short form are widely used to assess all aspects of sexual function but are not specific for urethral stricture disease and the short form does not evaluate ejaculation (4). Coursey et al evaluated sexual function after urethroplasty in 200 patients with a validated PROM and found a 30% rate of erectile dissatisfaction based on type of urethroplasty with higher rates noted in the penile flap cohort, but concluded that the rate was similar to those who underwent circumcision (5). The Brief Male Sexual Function inventory (BMSFI) was developed as an assessment tool for male sexual health but is not specific for urethral stricture disease (6). The Men's Health Sexual Questionnaire (MHSQ) is a validated questionnaire (7) and has been employed to assess ejaculatory function following urethroplasty (8) but again is not urethral stricture specific. Patel et al have specifically employed the MHSQ ejaculatory domain to assess sexual function following buccal graft for staged penile urethroplasty and reported minimal differences in erectile and ejaculatory function but reported definite changes in penile curvature, length and sensitivity (9). Barbagli et al. have developed a non validated PROM to assess the effect of bulbar urethroplasty on sexual function and reported no erectile dysfunction post operatively but 23% of patients studied reported ejaculatory dysfunction (10).

Historically, a successful outcome following urethroplasty was based on the need for secondary intervention to improve outflow symptoms but outcomes should be all inclusive and evaluate psychological, voiding and sexual outcomes as well as quality of life with a specific, validated questionnaire.

Breyer et al have published preliminary data on the development of a patient specific outcome measure, the 32 item Urethral Stricture Symptoms and Impact Measure (USSIM), which will further address both voiding and sexual symptoms following urethroplasty (1, 11).

Undoubtedly, as the assessment (12) of urethral stricture disease becomes more elaborate measuring all outcomes following urethroplasty will become routine practice for those Urologists offering urethral reconstruction. The authors are to be commended on highlighting the lack of validated questionnaires currently available to those working in the field of urethroplasty.

Yours Sincerely,

Authors
